# Reducing Resident Physician Workload to Improve Well Being

**DOI:** 10.7759/cureus.5039

**Published:** 2019-06-29

**Authors:** Mejalli Al-kofahi, Ghulam Rehman Mohyuddin, Melissa E Taylor, Leigh M Eck

**Affiliations:** 1 Internal Medicine, University of Kansas Hospital & Medical Center, Kansas City, USA

**Keywords:** graduate medical education, swing shift, internal medicine, resident wellness, intermediate shift, resident well being, residents workload, workload

## Abstract

Introduction

Resident physician’s well-being has been postulated to worsen with longer shifts. At our institution, the admitting physician evening shift (known as short call) had been associated with higher levels of stress and reduced well-being among residents due to longer work hours and an excessive number of admissions. We introduced an intermediate swing shift to help mitigate those effects. This study sought to assess the outcomes of introducing the swing shift on the timeliness of leaving the hospital for the short call physician, and the median number of admissions done by the short call, swing shift, and night shift resident physicians.

Method

The swing shift was designed to cover admitting duties from 4:00 to 11:00 pm on weekdays, with support from both the short call and night shift resident physicians. Internal Medicine residents in their second or third year of training and combined Medicine/Psychiatry residents in their third, fourth or fifth year of training, were surveyed prior to the implementation of the swing shift and four-months post-implementation. Time of leaving the hospital and number of admissions before and after the introduction of the swing shift were compared. Data were recorded as frequencies and presented as medians.

Results

There were 27 surveys completed prior to swing shift implementation and 43 surveys completed post-implementation with a response rate of 52% and 83%, respectively. Surveys post-implementation were divided into 29 for the short call shift survey, six for the swing shift survey, and eight for the night shift survey. Residents who did not perform the short call physician duties were excluded, limiting the prior to implementation surveys from 27 to 25 and the post-implementation short call surveys from 29 to 19. Prior to swing shift implementation, the median time of leaving for the short call physician was 8:30 to 9:00 pm; the median number of admissions were four and eight, done by short call physician and night shift physician, respectively. Whereas post-swing shift implementation, the median time of leaving for short call physician was 7:00 to 7:30 pm, and for swing shift physician was 11:30 pm to midnight. The median number of admissions were two, five, and five done by the short call, swing shift, and night shift physicians, respectively. All residents reported the swing shift allowed them to take better care of patients and follow up on their tasks.

Discussion and conclusion

Delayed resident physicians departure at the end of their respective shift was associated with extended shifts. It is thought to be caused by an increased number of admissions, late shift admissions, and time of day shift with 4:00 to 9:00 pm being the busiest. The addition of the swing shift increased the ability of the short call resident physician to leave the hospital at the end of their shift and reduced the median number of admissions done by the short call and night shift resident physicians, hence likely improving resident’s well-being while preserving the total number of admissions.

## Introduction

Physician well-being is an increasing focus in the medical community. Increased workload burden and long work hours amongst residents can be contributing factors behind increased stress. The Accreditation Council for Graduate Medical Education (ACGME) proposed the 80-hour work limit to improve patient safety, resident physician’s well-being, and education. It included daily limits on work hours for residents with the required time off between clinical duties [1]. Since then, most residency programs became highly dependent on assigning residents to various shifts in order to implement those restrictions [2]. There is yet to be a true consensus on the overall impact of such changes [3]. However, a significant proportion of systematic reviews pointed towards improved resident’s well being with reduced work hours and shorter shifts [2,4-6]. In ACGME surveys, house-staff meetings, semi-annual reviews with faculty, and liaison committee meetings, at our Internal Medicine residency program, the residents often cited one particular two-hour shift at the Veterans Affairs (VA) hospital as one to be associated with high workload burden, leading to prolonged shift hours and reduction in well-being.

The resident body in our program is comprised of 109 residents, including 69 categorical residents, 10 combined medicine/psychiatry residents, and 30 preliminary residents. The structure is based on a three plus one model, where residents have a maximum of three weeks of an inpatient rotation in a row with one week of continuity and specialty clinic. About 30% of the residents training is spent at the VA, with six academic inpatient teams, each consisting of one senior resident and two interns. Senior residents perform clinical duties from 6:00 am to 5:00 pm for their block of three weeks on those academic inpatient teams, during which they take short call shifts, where the end of shift time is extended to 7:00 pm to provide cross coverage and allow other team members to leave at 5 pm. Up to six of those short call shifts could include expanded clinical duties when those senior residents are assigned the role of the Medical Officer of the Day (MOD).

The MOD is a senior resident, postgraduate year (PGY) two or higher, who is responsible for accepting and triaging medicine admissions and consults in the hospital. This resident’s involvement in the admission process and other clinical expectations depends on the day of the week and the time of day. On weekdays, the MOD duties are performed by one senior resident from 8:00 am to 5:00 pm, during which that resident personally does all new medicine admissions from 8:00 am to noon, then triages and delegates admissions to the teams from noon to 5:00 pm. This resident is expected to evaluate medicine consults, and respond to code situations, but is not otherwise involved in the care of already hospitalized patients. From 5:00 pm to 7:00 pm, a senior resident who is on his/her team’s short call shift assumes the MOD role and is referred to as the short call MOD. Responsibilities include; continuing care of their own team, performing admissions to their team, triaging to all other teams, supervising all admissions done by interns, and assisting in the care of all floor and intensive care units (ICU) patients when more junior residents or interns need assistance. From 7:00 pm to 8:00 am the next day, a third senior resident assumes the role of the MOD and is expected to perform all overnight admissions and assist in the care of floor and ICU patients with the aid of two overnight interns. This resident is referred to as the night shift MOD.

Resident physicians reported that the short call MOD responsibilities often extended far beyond the designated end of shift time which caused significant distress and decrease in overall well-being at the VA. To mitigate this, an intermediate swing shift was introduced, overlapping with the short call MOD shift and the night MOD shift during periods of high admission burden and transition of care. This study sought to examine the effects of introducing the swing MOD shift on the timeliness of leaving the hospital for the short call MOD, and the median number of admissions for the short call, swing shift, and night MODs. It also assessed the median time at which the swing MOD left the hospital and the impact of such shift on patient care, via subjective reporting by the short call and night MODs.

## Materials and methods

This was a survey-based study of senior Internal Medicine and combined Medicine/Psychiatry residents, conducted during the 2017-2018 academic year. The introduced intermediate swing MOD shift was comprised of one senior resident, who performed the same MOD duties as short call and night shift MODs from 4:00 pm to 11:00 pm on weekdays. The swing shift MOD took first call on admissions with support from both the short call and the night MODs, each in their designated shift time. The resident on this shift did not have any other obligations prior to his/her shift on weekdays, or on the weekends, and was only required to do this shift for five days in a row, with two days off. The swing shift was introduced into the resident's schedule instead of an inpatient renal transplant service at the University hospital that was discontinued due to low census. Inclusion criteria were Internal Medicine PGY two or three residents and combined Medicine/Psychiatry PGY three, four, or five residents who had done at least one short call MOD shift at the VA prior to implementation. 

The study population was divided into pre- and post-swing shift implementation. Prior to swing shift implementation, one survey was obtained to establish the control group. Four months post-swing shift implementation, three separate surveys were obtained on three resident populations including, short call, swing, and night MODs. The pre-swing shift survey and the post-swing shift short call MOD survey had shared parameters that included PGY level, number of shifts done, time of leaving at the end of shift, and number of admissions during shift. The pre-swing shift implementation survey had residents choose two of five contributing factors for longer short call MOD shifts and asked to rate them as primary or secondary factors (Table 1). The post-implementation short call MOD survey included a true/false question asking residents if having a swing shift MOD helped take better care of the patients on their teams and allowed time to follow up on day-time tasks. Post-implementation swing MOD survey parameters were PGY level, number of admissions, and time of leaving. Whereas post-implementation night MOD survey parameters were PGY level, number of admissions before and after swing shift implementation, and rating the helpfulness of swing shift on a scale of one to five, with five being "Great" support.

**Table 1:** Contributing factors for longer short call MOD shifts as designated by the pre-implementation short call MOD shift survey. Arranged in descending order from most cited to least MOD: Medical Officer of The Day

Contributing factors for longer short call MOD shifts
Number of admissions during MOD shift
Significant requirement for intern supervision
Rapid responses, codes, or other emergent patient care
Supervision of daytime admissions not supervised or staffed by daytime team
Volume of clinical work

RedCap was used to design the surveys that were collected anonymously from the targeted populations. Data were collected as frequencies and presented as medians, compared using paired t-test as it is a paired sample with normal distribution assumed. Analysis of variance was conducted on the continuous variables using SPSS version 24. A P value less than 0.05 was considered statistically significant.

## Results

**Figure 1:**
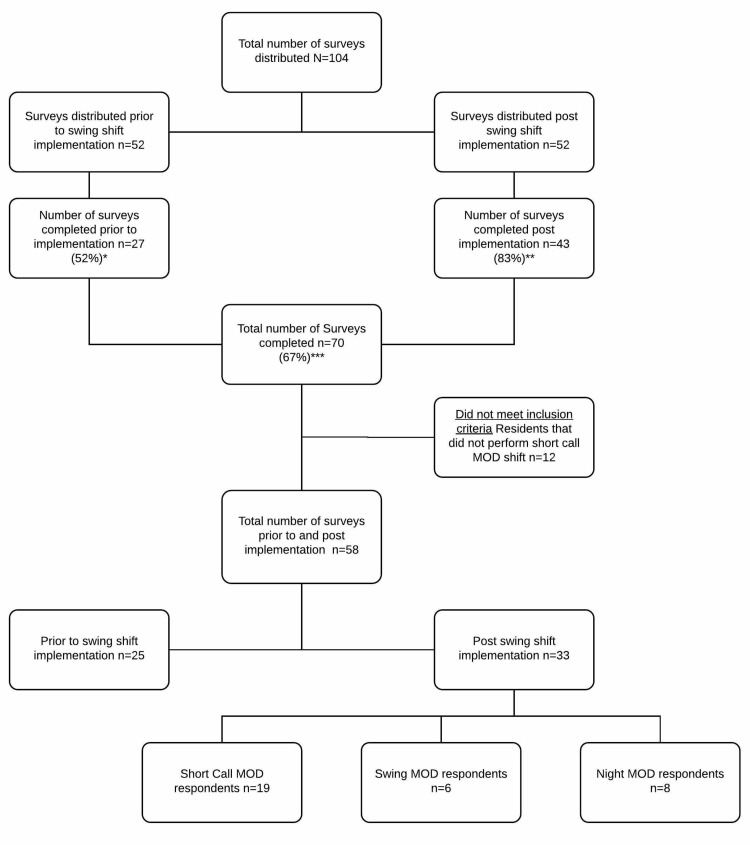
Number of surveys distributed divided into the two main populations of interest; prior to and post-swing shift implementation, with response rates reported. The post-implementation surveys are further delineated into short call MOD, swing MOD, and night MOD surveys. MOD: Medical Officer of The Day *This percentage represents the survey's response rate prior to swing shift implementation with 27 out of 52 completed. **This percentage represents the survey's response rate post swing shift implementation with 43 out of 52 completed. ***This percentage represents the survey's total response rate prior to and post-swing shift implementation with 70 out of 104 completed.

There were 52 residents who met the primary inclusion criteria of being Internal Medicine PGY two or three residents or combined Medicine/Psychiatry PGY three, four, or five residents. Each resident was surveyed prior to and post implementation giving a total number of 104 surveys sent. Out of which a total of 70 surveys were completed (67% response rate), 27 prior to swing shift implementation (52% response rate), and 43 post-implementation (83% response rate). Twelve surveys were excluded, as respondents did not perform the short call MOD duties, divided into two pre-swing shift surveys and 10 post-swing shift short call MOD surveys limiting those surveys numbers from 29 to 25 and from 29 to 19, respectively. The post-swing shift surveys were divided into 19 short call MOD surveys, six swing MOD surveys, and eight night MOD surveys (Figure 1).

Median PGY level was PGY three or greater for pre-implementation short call MOD and post-implementation swing MOD, and PGY two for post-implementation short call MOD and night MOD. The median number of short call MOD shifts done pre and post-swing shift implementation were eight, and four respectively (p = 0.03). The median time of leaving the hospital for the short call MOD was 8:30 to 9:00 pm pre-implementation, and 7:00 to 7:30 pm post-implementation (p = 0.0001). The median number of admissions during short call MOD shift pre and post-implementation was four and two, respectively (p = 0.0001) (Table 2). Most cited primary contributing factors for longer short call MOD shifts was "number of admissions during MOD shift", whereas most cited secondary factor was "significant requirement for intern supervision". All residents who returned the post-implementation short call MOD survey answered true to the true/false question assessing if the swing MOD shift helped them take better care of the patients on their teams’ and allowed time to follow up on daytime tasks. 

**Table 2:** Pre- and post-implementation short call MOD surveys comparison, with reduction in time of leaving at the end of shift from 8:30-9:00 pm pre-implementation to 7:00-7:30 pm post-implementation, and number of admissions during MOD shift from four pre-implementation to two post-implementation. MOD: Medical Officer of The Day

Parameter (median)	Pre-implementation Short call MOD	Post-implementation Short call MOD	p value
Time of leaving at the end of shift	8:30-9:00 pm	7:00-7:30 pm	p=0.0001
Number of admissions during MOD shift	4	2	p=0.0001

Post-implementation swing MOD surveys showed a median number of admissions of five and a median time of leaving of 11:30 pm to midnight. The median number of admissions during night MOD shift was eight prior to swing shift introduction and five afterward (p = 0.005). Seven out of the eight night MOD survey respondents rated the helpfulness of the swing shift at five with one resident rating it at three out of five.

## Discussion

This study sought to assess the outcomes of introducing an intermediate swing shift on the short call MOD ability to leave the hospital on time, and the median number of admissions done by the short call, night, and swing MODs. It also assessed the median time at which the swing MOD left the hospital and the impact of this shift on patient care. Post-swing shift implementation, the short call MOD median time of leaving the hospital matched the designated end of shift time. The overall number of admissions was not affected but was distributed between three MODs instead of two. The median number of admissions done by the short call MOD and the night MOD was reduced by two and three, respectively. Whereas, the swing MOD median number of admissions was five. The swing MOD residents had a 30-60-minute delay in their time of leaving. With the lesser burden of admissions that the swing shift provided, all residents reported a perceived better ability to care of patients and to follow up on daytime tasks. 

To our knowledge, this was the first study to assess the effects of introducing an intermediate shift to improve resident’s well-being by improving the timeliness of hospital departure and reducing the number of admissions. A negative association between delayed residents departure at the end of a shift and the resident’s well-being had been established by various systematic reviews of observational and opinion surveys [2,4-6]. Reasons behind delayed residents’ departure have also been explored before and three major culprits were found; increased overall number of admissions during the shift, increased number of late shift admissions [7-8], and time of day for the shift, with the busiest time for admissions being from four to nine pm [9]. This was consistent with the reported effects on the short call MOD shift, as the reduction of the total number of admissions and the extra support provided by the swing MOD in the busiest time for admissions, resulted in an increased ability of residents to leave the hospital at the designated end of shift. Thus, by improving the timeliness of hospital departure and reducing the number of admissions we perceive an indirect improvement in resident’s well-being.

Dependence on shift work and night float have been increasing in residency programs across the nation. The impact of this on resident’s education is not well established [2-3]. Therefore, the effects of the addition of the swing shift on education require further assessment. One speculation is that it may lead to reduced learning opportunities [10]. In our study, the number of admissions done by each MOD was reduced, and this could be argued to cause reduced learning opportunities. However, the total number of admissions among the three MODs remained the same, thus the educational opportunities were more evenly spread which may allow each experience to be more meaningful and better poised for learning.

Future directions would need to consider the exact parameters of improved resident’s well-being, such as chronic fatigue, sleep deprivation and rates of burnout. Previous studies suggested worsening burnout manifested as emotional exhaustion and depersonalization with longer work hours [11]. Others have suggested an increased propensity for medical errors and reduced performance of medical trainees due to fatigue and sleep deprivation [12]. Although the addition of the swing shift did add an intermediate third resident with an additional handoff done between that resident and the night MOD, all residents reported a perceived better ability to take care of patients and follow up on daytime tasks.

This study was conducted to assess the effects of the swing shift only after four months of implementation on a relatively small number of residents in one single institution. Results were obtained via surveys that depended on residents answering from memory after the end of their shift; thus, recall bias is a concern. However, the surveys were administered as close as possible after shift implementation to minimize this as much as possible.

## Conclusions

Introducing an intermediate swing shift that overlaps with the short call MOD shift and the night MOD shift led to improved ability of the short call MOD to leave at time and reduced number of admissions per shift. Both effects were hypothesized to improve overall resident’s well-being while maintaining equal educational opportunities. Actual parameters of improved resident’s well-being need further assessment as well as the impact of adding additional handoff to the workflow at the VA hospital.

## References

[1] Nasca TJ, Day SH, Amis ES, ACGME Duty Hour Task Force (2010). The new recommendations on duty hours from the ACGME Task Force. N Engl J Med.

[2] Reed DA, Fletcher KE, Arora VM (2010). Systematic review: association of shift length, protected sleep time, and night float with patient care, residents’ health, and education.. Ann Intern Med.

[3] Bolster L, Rourke L (2015). The effect of restricting residents’ duty hours on patient safety, resident well-being, and resident education: an updated systematic review.. J Grad Med Educ.

[4] Institute of Medicine (2019). Resident Duty Hours: Enhancing Sleep, Supervision, and Safety. Washington, DC: The National Academies Press..

[5] Philibert I, Nasca T, Brigham T, Shapiro J (2012). Duty-hour limits and patient care and resident outcomes: can high-quality studies offer insight into complex relationships?. Annu Rev Med.

[6] Fletcher KE, Reed DA, Arora VM (2011). Patient safety, resident education and resident well-being following implementation of the 2003 ACGME duty hour rules. J Gen Intern Med.

[7] Arora VM, Georgitis E, Siddique J, Vekhter B, Woodruff JN, Humphrey HJ, Meltzer DO (2008). Association of workload of on-call medical interns with on-call sleep duration, shift duration, and participation in educational activities. JAMA.

[8] Gonzalo J, Herzig S, Reynolds E, Yang J (2012). Factors associated with non-compliance during 16-hour long call shifts.. J Gen Intern Med.

[9] McCoy CP, Halvorsen AJ, Loftus CG, McDonald FS, Oxentenko AS (2011). Effect of 16-hour duty periods on patient care and resident education. Mayo Clin Proc.

[10] Charap M (2019). Reducing resident work hours: unproven assumptions and unforeseen outcomes. Ann Intern Med.

[11] Parshuram CS, Amaral ACKB, Ferguson ND (2015). Patient safety, resident well-being, and continuity of care with different resident duty schedules in the intensive care unit: a randomized trial.. CMAJ.

[12] Parshuram CS, Dhanani S, Kirsh JA, Cox PN (2004). Fellowship training, workload, fatigue and physical stress: a prospective observational study.. CMAJ.

